# Cook It Up! A community-based cooking program for at-risk youth: overview of a food literacy intervention

**DOI:** 10.1186/1756-0500-4-495

**Published:** 2011-11-15

**Authors:** Heather MC Thomas, Jennifer D Irwin

**Affiliations:** 1 Middlesex-London Health Unit, 50 King Street, London, Ontario, Canada, N6A 5L7; 2 The University of Western Ontario, London, Ontario, Canada, N6A 5B9

## Abstract

**Background:**

In Canada, there are limited occasions for youth, and especially at-risk youth, to participate in cooking programs. The paucity of these programs creates an opportunity for youth-focused cooking programs to be developed, implemented, and evaluated with the goal of providing invaluable life skills and food literacy to this potentially vulnerable group. Thus, an 18-month community-based cooking program for at-risk youth was planned and implemented to improve the development and progression of cooking skills and food literacy.

**Findings:**

This paper provides an overview of the rationale for and implementation of a cooking skills intervention for at-risk youth. The manuscript provides information about the process of planning and implementing the intervention as well as the evaluation plan. Results of the intervention will be presented elsewhere. Objectives of the intervention included the provision of applied food literacy and cooking skills education taught by local chefs and a Registered Dietitian, and augmented with fieldtrips to community farms to foster an appreciation and understanding of food, from 'gate to plate'. Eight at-risk youth (five girls and three boys, mean age = 14.6) completed the intervention as of November 2010. Pre-test cooking skills assessments were completed for all participants and post-test cooking skills assessments were completed for five of eight participants. Post intervention, five of eight participants completed in-depth interviews about their experience.

**Discussion:**

The *Cook It Up! *program can provide an effective template for other agencies and researchers to utilize for enhancing existing programs or to create new applied cooking programs for relevant vulnerable populations. There is also a continued need for applied research in this area to reverse the erosion of cooking skills in Canadian society.

## Background

Poor dietary habits during adolescence (ages 13-18) have negative impacts on several health and wellness indicators including: day-to-day wellbeing and functioning; achievement and maintenance of healthy weights; proper growth and development patterns; and dental health [1-5]. Researchers have shown that when youth are involved in preparing food for meals, they are more likely to eat more nutrient-rich foods including higher intakes of fruits and vegetables, higher intakes of key nutrients, and lower intakes of fat [6-11]. However, these studies assume youth have access to quality food on a regular basis and live in a family-style environment. What is less evident in the literature is youth involvement in food-related tasks such as food shopping and preparation [12-14], especially when the priority population is at-risk youth in transition from the family home or foster care to independent living. It is challenging to find a comprehensive definition of at-risk youth; however, there is agreement in the literature that "at risk youth" can include characteristics such as: diverse racial backgrounds; negative influence from family, environment or peers; social factors that restrict healthy mental and social growth; limited financial resources; difficulty achieving optimal education; and behavioural issues [15-17]. Any and all of these characteristics can make it difficult for the at-risk youth to become a successful adult [15]. These youth are particularly important to focus on, given their increased vulnerability and thus greater difficulty in achieving the social determinants of health [18] which are influenced by: social gradient; stress; early life; social exclusion; social support; addiction; and food [19].

While an "official" definition for food literacy is not presented in the literature, it can be defined as the ability to make healthy food choices by having the skills and knowledge necessary to buy, grow, and cook food [20] with implications for improving health [21]. Food and cooking skills/food preparation are important for several reasons related to health, knowledge, empowerment, engagement, culture, food security, and fun [22-25]. An engaging cooking skills program targeting youth builds **se**lf-efficacy, food knowledge and literacy, self-confidence, and self-esteem, while potentially improving the social determinants of health [26]. Cooking skills programs are effective and necessary interventions [26] which not only provide the opportunity for practical application of life skills, but also the opportunity for social connectedness among peers and role modelling for career planning and potentially higher level education. The manuscript provides information about the process of the program planning, the intervention implementation, and the evaluation plan for *Cook It Up! *Results of the intervention will be presented elsewhere. *Cook It Up! *was a community-based cooking program for at-risk youth implemented in May 2009 and concluding in February 2011. This paper will inform future program planners and researchers in the creation, development, implementation, and evaluation of similar cooking skills programs.

## Methods

### Program Planning Process for Cook It Up!

*Cook It Up! *was an 18-month community-based cooking program for at-risk youth that focussed on food education and building cooking skills. The London Community Resource Centre was the host agency for *Cook It Up! *Locally, there were no other evaluated cooking programs for youth. The primary objective of *Cook It Up! *was to provide education and to increase skills and awareness of agriculture, healthy eating, food preparation, and food purchasing skills among London, Ontario youth. This objective was accomplished by introducing youth to the local Ontario agricultural industry and building new and essential life skills through cooking classes. With the erosion of cooking skills among youth [23,27,28], this intervention was aimed at enhancing existing proficiency and building greater cooking competence and food literacy among this population.

*Cook It Up! *provided youth-centred, hands-on food literacy education that highlighted general nutrition, food safety, selection, preparation, and cooking skills. Agriculture fieldtrips showcased seasonal Ontario-grown food commodities which enhanced the participants' learning about locally grown foods. Local chef facilitators targeted, coordinated, and implemented activities within each cooking and fieldtrip session relevant to the needs and desires of the youth. Effort was taken to build upon existing skills each week in order to improve skills while empowering the youth by building upon their food-related self-esteem and self-efficacy. Baseline skills were measured using a previously developed and piloted pencil-and-paper administered pre-test cooking skills assessment questionnaire [26].

## Findings

### Steering Committee Selection

The Steering Committee was a necessary consideration to assist in successful program development. The funding agency required *Cook It Up! *to engage in new or non-traditional community partners with interest in promoting the local agri-food industry and the public health benefits of Ontario grown products. With this requirement in mind for program planning, key stakeholders were selected to direct the project. The Steering Committee was comprised of 10 individuals and included membership of local chefs (for cooking skills education) local farmers (for the connection to local agri-food industry), education specialists (for guidance about how to work with at-risk youth), social service agency representatives focusing on the youth population (to assist in participant recruitment and youth engagement), public health representatives (to assist in proposal writing, research, evaluation, and nutritional aspects of the initiative), food service industry representatives (to provide opportunities for fieldtrips), academic representatives (to assist with research and evaluation), community members with interest and skills in this project and/or priority group (to ground the Steering Committee and ensure best interests of the participants and program goals were prevalent), and a food specialty store owner (to provide business representation and program resources).

### Program Coordinator Selection

Equally important to the Steering Committee recruitment was the recruitment and selection of the Program Coordinator. The Executive Director of the host agency for *Cook It Up! *met the Program Coordinator at a community meeting where food literacy was discussed. He was invited to serve in this role in the *Cook It Up! *program because he had worked in the food service industry and shared a passion for local food, youth education, and cooking. His greatest strength was his existing connections to local chefs, farms, and farmers' markets. The Program Coordinator's role was to engage and build rapport with local chefs, farmers, and farmers' markets to ensure broad and diverse opportunities for cooking sessions and fieldtrips.

### Chef and Volunteer Recruitment

The Steering Committee promoted the *Cook It Up! *program and its need for guest chef involvement at a local chefs' association meeting. Chefs in this association were provided an overview of the initiative and were encouraged to become involved in some capacity, either by providing a cooking demonstration and skill session with the youth or assisting with the Steering Committee in whatever capacity they chose. In addition to this method of chef recruitment and selection, the Program Coordinator reviewed the proposed "menu" of cooking skills and seasonal availability of local produce, and paired local chefs with particular interest and/or skill in certain cooking methods and recipes.

The Steering Committee had a strong connection to the University of Western Ontario and the Foods and Nutritional Sciences program at Brescia University College (BUC). One of our Steering Committee members, and also a professor at BUC, promoted *Cook It Up! *volunteer opportunities with her students, four of whom became involved in the program as part of the community placement component of their course. The Public Health Dietitian from the Middlesex-London Health Unit supervised three graduate students who participated as volunteers with *Cook It Up! *and also contributed to proposal writing, research, and program content development.

The Steering Committee recommended that volunteers with a specific background working with at-risk youth be recruited. One volunteer was a retired teacher who specialized in working with children with special needs. Her background, patience, problem-solving strategies, and general demeanour with the participants in *Cook It Up! *was an ideal combination when working with youth who were easily distracted, demonstrated behavioural issues, and with whom were, at times, difficult to connect. Additionally, the Steering Committee recruited a teacher with expertise in secondary school family studies/food and nutrition curriculum as a volunteer. The volunteers' roles and responsibilities were to keep the participants on track in terms of completing preparation and cleaning tasks, help participants navigate through the fieldtrip when independently completing assigned tasks (e.g., collecting produce from the field such as apple picking, grocery shopping), assist the participants in the completion of their weekly "journals," which summarized the youths' weekly involvement, monitor safety issues in the kitchen, remind participants to be safe, clean, and organized, ensure cooking and field trip sessions ran smoothly, and assist the Program Coordinator or chefs in any way required. The volunteers recruited were very positive about the program; however, some of them had never worked with at-risk youth in the past. For this reason, it was necessary to implement sensitivity training. We engaged one qualified member of our Steering Committee members to facilitate sensitivity training for all volunteers.

### Target Population and Recruitment Strategies

The *Cook It Up! *program involved a significant time and participation commitment; therefore, the Steering Committee wanted to ensure the participants involved in this pilot project were fully committed to the program. To recruit potential youth participants, the Steering Committee utilized local media outlets to introduce the program to the community. Key Steering Committee members were interviewed in local newspapers and on television programs. The initiative was also promoted on local agencies' websites, on social media outlets such as Facebook^® ^and YouTube^®^, and via word of mouth. An online and paper application form was available for potential participants. Interested parties were directed to the London Community Resource Centre's website to learn more about the program and complete the application form. In addition to the application form, the potential participants met with members of the Steering Committee who conducted informal interviews with the youth to determine fit, interest, enthusiasm, and commitment to the program. This proved to be an effective recruitment and retention strategy. Originally, 30 youth applied to the program, but through self-selection out of the program due to a variety of different reasons (e.g., time commitment, program components, conflicts with other activities) and the interview process with Steering Committee members, the final number of participants in *Cook It Up! *was nine. There was attrition of one participant due to personal issues. The other eight participants remained for the entire duration of the program (18 months).

At times, at-risk youth in the program presented with a variety of behavioural problems which negatively influenced the learning environment, as described in the literature [29]. This negative behaviour created frustration among volunteers, chefs, and other participants. In these circumstances, volunteers used their sensitivity training to mediate the situation, reduce frustration, and keep the program on track. In each cooking and fieldtrip session, there were eight participants, a minimum of four volunteers, the Program Coordinator, a chef from the Steering Committee, and guest chef. For this program, a maximum of 15 people, including participants, the Program Coordinator, chef(s) and volunteers, was desirable. Careful consideration of the priority group selected and their unique needs determined the number and expertise of volunteers at each session.

### Program Components and Implementation Process

#### Cooking Component

Youth participants attended *Cook It Up! *for cooking sessions twice monthly from August 2009 to November 2010 on Mondays between 4:00 and 6:00 pm. During the 18-month program, youth engaged in a variety of cooking opportunities focusing on seasonal and local food ingredients and the sessions were facilitated by local chefs. Each session consisted of the Program Coordinator outlining the recipe for the session and introducing the guest chef who would be working with the youth. The chef taught participants skills necessary to complete the selected recipes. Each session featured an overview of the historical context of the foods chosen to create the recipe in service of educating the youth about the origins of foods. A variety of recipes was introduced but effort was taken to ensure that the skill required to perform the execution of each recipe also incorporated skills that had been used previously, thus building upon the youths' development of their cooking proficiency from week to week. This approach was an effective way to empower the youth participants because they gained confidence and pride in their cooking abilities. Youths' gratification with their skills was shared with a broader audience with the publication of a cookbook highlighting the recipes used in the program and giving the reader a glimpse into the fieldtrip experiences through the eyes of the participants. Though not a planned component of the program, the cookbook provided a summary of the recipes used and fieldtrips experienced over the course of the program and generated some modest funds for the initiative. The cooking sessions took place at a centrally located faith-based organization with excellent kitchen facilities which were approved by the local health unit. The Minister of the faith-based organization was amenable to having the *Cook It Up! *program utilize these facilities and an excellent partnership was developed as a result.

#### Fieldtrip Component

The participants engaged in fieldtrips to local farms and farmers' markets once monthly. Fieldtrips were selected to connect the youth to their cooking experiences. For example, specific farms growing particular commodities were selected given their produce could complement the recipes well. In the spring, a trip to a local sugar bush to learn how maple syrup was made complemented the cooking session on pancakes. A fall fieldtrip to a local dwarf apple tree orchard led to recipes for applesauce, apple pie, apple crisp, and homemade pie crust.

In addition to "food" related fieldtrips, other fieldtrips were provided. An opportunity to expand the participants' appreciation for formal culinary education resulted in a fieldtrip to the local community college. Youth in the *Cook It Up! *program were invited to visit the college-level culinary program and observe a food demonstration in the test kitchen, learn about the culinary programs available at the post-secondary institute, and speak to the first year Coordinator of Chef Training and Culinary Management in the School of Tourism and Hospitality. This fieldtrip inspired some of the youth to seriously consider post-secondary school education in this field as a future academic goal.

There were two additional opportunities that were presented to the *Cook It Up! *program throughout the duration of the intervention that further developed participants' cooking skills. First, the group was asked to cater the inauguration of a neighbourhood community centre. The group prepared recipes, served the food, and enjoyed the rewards of positive feedback from the 100 guests who attended. The second event was a catering opportunity for a local youth club during National Youth Week. Again, the participants organized, prepared, and served recipes they had previously made to approximately 50 guests. Both opportunities allowed the youth to perform the tasks required with confidence and enthusiasm.

For all cooking components and fieldtrip activities of *Cook It Up!*, participants were included in the development of the session thus encouraging their engagement and participation. Youth engagement was an important approach implemented in *Cook It Up! *and ensured the intervention was meeting the needs of the participants at all times.

#### Program Cost

There was no cost to youth to participate in the *Cook It Up! *program. Costs associated with the operation of the program including food, transportation to cooking sessions and fieldtrip locations, basic kitchen equipment for youth (provided to them at the end of the program), and other incidental fees were included in the grant budget.

### Program Evaluation Plan

A pre/post cooking skills assessment questionnaire was used to monitor participants' progress and allow for a baseline comparison. This design also provided preliminary evidence for the future development of cooking skills assessment for at-risk youth or other populations. Additionally, qualitative interviews were undertaken to determine the effectiveness of the program from the perspective of all participants involved (e.g., Steering Committee members, chefs, volunteers, parents/guardians, and youth). Also, Photovoice methodology [30] was introduced to determine the youths' perceptions of the barriers and facilitators of cooking skills development external to the *Cook It Up! *program.

According to Bandura [31], one's perceived ability to perform behaviours, self-efficacy, is enhanced when one has the practical and necessary skills for completion of the task and/or behaviour. The provision of this hands-on, practical life skills program in order to build self-efficacy, knowledge, self-confidence, and self-esteem was a unique intervention for at-risk youth. *Cook It Up! *was designed to provide participants with life skills necessary for leading a responsible, independent life (e.g. food selection, shopping, preparation, and food preservation skills as well as decision-making, communication, and social skills) which served to enhance their self-efficacy. Offering the program was only the first step; without knowing participants' receptiveness to and experiences with the program it is difficult to identify whether it should continue, be expanded, or if it had any unanticipated negative effects. Therefore, a formative evaluation was implemented to assess the *Cook It Up! *program to determine its value from the perspective of participants, as well as what could be done to "assess the relevance, comprehension, and acceptability of activities, materials, methods," of the program [32].

The formative evaluation appraised the education and skill building initiative focusing on nutrition, food safety, food preparation and cooking skills, and agriculture fieldtrip experiences to a variety of local farms. The research qualitatively assessed participants' (youth community partners', and parents'/guardians') experiences with *Cook It Up! *over the 18-month period. The objectives of the formative evaluation were three-fold. First, the evaluation assessed the strengths and weaknesses of the program and its delivery. Secondly, we anticipated uncovering obstacles, barriers or unexpected opportunities that made the program more effective. Finally, this evaluation generated understandings about how the program content and implementation could be improved. Verbal informed consent was received from research participants. A purposeful sample of participants was sought for the in-depth interviews to maximize the richness of information obtained pertinent to the research question. Interviewing continued until interpretation of the interviews revealed no new significant insights, thus attaining data saturation. It was estimated that between 10 and 20 in-depth interviews would be necessary before data saturation was realized [33]. A total of 25 participants were interviewed for the formative evaluation (3 guest chefs, 5 Steering Committee members, 3 fieldtrip operators, 6 volunteers, 3 parents/guardians, and 5 at-risk youth participants). There was excellent response by participants to assist with the research; therefore, the lead investigator allowed all interested participants to contribute. Saturation was reached at 19 interviews. Six additional interviews were conducted to ensure nothing was missed and to accommodate participants willing to support the research. The research facilitated the development of a "how-to" community resource manual available for local and provincial distribution. The manual was pre-determined as a "deliverable" to the main funding agency of the project (Ontario Agri-Food Education, Inc.).

The final research project for *Cook It Up! *was a Photovoice project which qualitatively assessed the barriers and facilitators youth participants experienced with respect to the development of healthy cooking skills in their environments peripheral to the *Cook It Up! *program. Verbal informed consent was received from research participants. Participants also took part in a discussion group to discuss the photographs taken and select the ones that best exemplified their perceived barriers and facilitators to the progression of their cooking skills. Upon completion of the project, participants were invited to share their photographs at a local art display/gallery, to showcase their work and involvement in the project.

For all research components of *Cook It Up!*, ethical approval was obtained from the Office of Research Ethics at the University of Western Ontario.

## Discussion

Our eating and meal preparation culture is changing. Domestic cooking skills are in a state of erosion, or at the very least, are in transition, such that the types of foods people cook, or perhaps more accurately "reheat," how they use food preparation skills, and where they cook are influenced by social, economic, and cultural contexts [34]. In most provinces in Canada, cooking skills are not taught in elementary schools and certainly taught much less in households today compared to the past [35]. Some researchers contend that domestic food preparation appears to be less pertinent to children and youth and there may exist a "de-skilling" of cooking resulting from the lack of introduction and opportunity to acquire cooking skills from parents, grandparents, or even school environments [34]. The limited awareness of food literacy, cooking skills, and knowledge about how foods are grown and harvested can create barriers to consuming a healthy diet [23].

On the upside, studies have demonstrated that hands-on cooking education has a very positive impact on behaviours and attitudes toward cooking and healthy eating such as increased consumption of fruits and vegetables, improved food safety behaviours, higher frequency of cooking, increased nutrition knowledge, higher self-efficacy, and less money spent on food [36-43]. Nutritional intake during the adolescent years impacts physical health, risk of future disease, and bodyweight [39]. However, there are few studies examining the food preparation and cooking skills of youth, especially at-risk youth. There are also few studies examining youths' understanding of the local agri-food industry and how it relates to their ability to select, prepare, cook, store, and enjoy foods prepared from "scratch." Cooking programs for youth with a focus on the local agri-food industry are an integral component of food literacy development to facilitate healthy lifestyles in this population.

### Implications for Practice

Cooking skills programs for youth provide numerous benefits, including the development of necessary life, social and economic management skills and education about healthy eating required for the promotion and enhancement of health. With the removal of food and cooking skills syllabi from school systems and narrow opportunities to learn food literacy in the home environment, there are limited opportunities for youth to learn and apply basic life- and food-related skills such as proper food selection, preparation, storage, and usage. Effective interventions that promote and foster cooking skills development targeting youth are needed. The *Cook It Up! *program provided a unique intervention to improve these important life skills in a population that is already at risk of experiencing challenges for many reasons.

This manuscript provides detailed applications of this community-based cooking intervention for other practitioners who want to create a similar program with their populations. Our hope is that the details provided enable other practitioners to build upon our work rather than creating an entire intervention from the ground up. It is through the sharing of this type of programmatic information that researcher-practitioners can co-create programs that will ultimately facilitate healthier food-related options among those in need.

## Competing interests

The authors declare that they have no competing interests.

## Authors' contributions

HMCT was in involved in all aspects of the development of the program, research design, data collection and analysis, and participated in the writing of the manuscript. JDI provided support and expertise related to the design and implementation of the study, provided revisions and comments to the manuscript, and approved the final version. Both authors read and approved the final version of the manuscript.

## References

[B1] Figueroa-Colon R Franklin FA Lee JY Aldridge R Alexander L Prevalence of obesity with increased blood pressure in elementary school-aged children South Med J 1997 90 806 13 10.1097/00007611-199708000-00007 9258307

[B2] Figueroa-Munoz JI Chinn S Rona RJ Association between obesity and asthma in 4-11-year-old children in the UK Thorax 2001 56 133 7 10.1136/thorax.56.2.133 11209102 PMC1745999

[B3] Fagot-Campagna A Emergence of type 2 diabetes mellitus in children: epidemiological evidence J Pediatr Endocr Met 2000 13 S6 1395 402 10.1515/jpem-2000-s613 11202215

[B4] Reilly JJ Methven E McDowell ZC Hacking B Alexander D Stewart L Kelnar CJH Health consequences of obesity Arch Dis Child 2003 88 748 752 10.1136/adc.88.9.748 12937090 PMC1719633

[B5] Serdula MK Ivery D Coates RJ Freedman DS Williamson DF Byers T Do obese children become obese adults? A review of the literature Prev Med 1993 22 167 77 10.1006/pmed.1993.1014 8483856

[B6] Anderson AS Bell A Adamson A Moynihan P A questionnaire assessment of nutrition knowledge - validity and reliability issues Public Health Nutr 2001 5 3 497 503 10.1079/PHNPHN2001307 12003663

[B7] Aumann M Briggs M Link N Emmerich Collett M Corrigan K Hart P Cuisine for Kids: a nutrition and culinary course for child nutrition program staff J Nutr Educ 1999 31 2 121 10.1016/S0022-3182(99)70408-5

[B8] Brown BJ Hermann JR Cooking classes increase fruit and vegetable intake and food safety behaviors in youth and adults J Nutr Edux Behav 2005 37 2 104 105 10.1016/S1499-4046(06)60027-4 15882491

[B9] Larson NI Story M Eisenberg ME Neumark-Sztainer D Food preparation and purchasing roles among adolescents: associations with sociodemographic characteristics and diet quality J Am Diet Assoc 2006 106 2 211 218 10.1016/j.jada.2005.10.029 16442868

[B10] Thonney PF Bisogni CA Cooking up fun! A youth development strategy that promotes independent food skills J Nutr Educ Behav 2006 38 5 321 323 10.1016/j.jneb.2006.03.007 16966055

[B11] Wrieden WL Anderson AS Longbottom PJ Valentine K Stead M Caraher M Lang T Gray B Dowler E The impact of a community-based food skills intervention on cooking confidence, food preparation methods and dietary choices - an exploratory trial Public Health Nutr 2007 10 2 203 211 17261231 10.1017/S1368980007246658

[B12] Hebert K Jacobson A Adolescent evening meal practices and attitudes toward the maternal role in evening meal preparation Intl J Consumer Stud 1991 15 249 259

[B13] Skinner J Salvettin M Penfield M Food intakes of working and nonworking adolescents J Nutr Educ 1984 16 164 167

[B14] Watt R Sheiham A Dietary patterns and changes in inner city adolescents J Hum Nutr Diet 1996 9 451 461 10.1046/j.1365-277X.1996.00480.x

[B15] Dobizl JK Understanding at-risk youth and intervention programs that help them succeed in school 2002 http://www2.uwstout.edu/content/lib/thesis/2002/2002dobizlj.pdf Retrieved March 29, 2011

[B16] Moore KA Defining the term "at risk." Child Trends 2006 http://www.childtrends.org/Files//Child_Trends-2006_10_01_RB_DefiningAtRisk.pdf Retrieved March 29, 2011

[B17] Sussman S Moran MB Sun P Pokhrel P Gunning M Kniazev V Masagutov R Peer group self-identification in samples of Russian and U.S adolescents J Drug Education 2010 40 2 203 215 10.2190/DE.40.2.g 21133332

[B18] Public Health Agency of Canada What makes Canadians healthy or unhealthy? 2003 http://www.phac-aspc.gc.ca/ph-sp/determinants/determinants-eng.php#unhealthy Retrieved March 29, 2011

[B19] World Health Organization Social Determinants of Health: The solid facts 2003 2 http://www.euro.who.int/__data/assets/pdf_file/0005/98438/e81384.pdf Retrieved August 16, 2011

[B20] The Food Literacy Project 2010 http://foodliteracyproject.org/Retrieved March 20, 2011

[B21] Begley A Gallegos D Should cooking be a dietetic competency? Nutr Diet 2010 67 41 46 10.1111/j.1747-0080.2010.01392.x

[B22] Anderson A Nutrition interventions in women in low-income groups in the UK P Nutr Soc 2007 66 25 32 10.1017/S0029665107005265 17343769

[B23] Lang R Caraher M Is there a culinary skills transition? Data and debate from the UK about changes in cooking culture HEIA J 2001 8 2 2 14

[B24] Lang R Caraher M Dixon P Carr-Hill R Cooking Skills and Health 1999 http://www.nice.org.uk/nicemedia/documents/cooking_skills_health.pdf Retrieved from on November 28, 2009

[B25] McLaughlin C Tarasuk V Kreiger N An examination of at-home food preparation activity among low-income, food-insecure women J Am Diet Assoc 2006 103 11 1506 1512 10.1016/j.jada.2003.08.022 14576717

[B26] Region of Waterloo Public Health Food skills of Waterloo region adults 2009 http://chd.region.waterloo.on.ca/en/healthylivinghealthprotection/foodskills.asp Retrieved from on October 19, 2011

[B27] Lai Yeung WT A study of perceptions of food preparation skills in Hong Kong adolescents HEIA J 2007 14 2 16 24

[B28] Short F Domestic cooking skills - what are they? HEIA J 2003 10 3 13 22

[B29] Sullivan CJ Childs KK O'Connell D Adolescent risk behaviour subgroups: An empirical assessment J Youth Adolescence 2009 39 5 545 562 10.1007/s10964-009-9445-5 19731001

[B30] Wang C Burris MA Photovoice: concepts, methodology, and use for participatory needs assessment Health Education & Behavior 1997 24 3 369 387 10.1177/109019819702400309 9158980

[B31] Bandura A Self-efficacy: toward a unifying theory of behavior change Psychology Review 1977 84 55 65 10.1037//0033-295x.84.2.191 847061

[B32] Green LW Kreuter MW Health Promotion Planning: An Educational and Ecological Approach 1999 Mountain View: Mayfield Publishing

[B33] Miller WL Crabtree B Hesse-Biber SN, Leavy P Depth Interviewing Approaches to qualitative research: a reader on theory and practice 2004 New York, NY: Oxford University Press 185 202

[B34] Caraher M Dixon P Lang T Carr-Hill R The state of cooking England: The relationship of cooking skills to food choices Brit Food J 1999 1 8 590 607

[B35] Dietitians of Canada Community Food Security: Position of Dietitians of Canada 2007 http://www.dietitians.ca/Dietitians-Views/Community-Food-Security.aspx Retrieved from on October 19, 2011

[B36] Crawford D Ball K Mishra G Salmon J Timperio A Which food-related behaviours are associated with healthier intakes of fruits and vegetables among women? Public Health Nutr 2007 10 3 256 265 17288623 10.1017/S1368980007246798

[B37] Cullen KW Watson KB Zakeri I Baronowski T Baronowski JH Achieving fruit, juice, and vegetable recipe preparation goals influences consumption by 4^th ^grade students 2007 http://www.ijbnpa.org/content/4/1/28 Retrieved from on November 28, 2009 10.1186/1479-5868-4-28 PMC1920529 17603875

[B38] Larson NI Perry CL Story M Neumark-Sztainer D Food preparation by young adults is associated with better diet quality J Am Diet Assoc 2006 106 12 2001 2007 10.1016/j.jada.2006.09.008 17126631

[B39] Larson NI Story M Eisenberg ME Neumark-Sztainer D Food preparation and purchasing roles among adolescents: associations with sociodemographic characteristics and diet quality J Am Die Assoc 2006 106 2 211 218 10.1016/j.jada.2005.10.029 16442868

[B40] Meehan M Yeh M Spark A Impact of exposure to local food sources and food preparation skills on nutritional attitudes and food choices among urban minority youth J Hunger Environ Nutr 2008 3 4 456 471 10.1080/19320240802529383

[B41] Shankar S Klassen A Influences on fruit and vegetable procurement and consumption among urban African-American public housing residents, and potential strategies for interventions Fam Econ Nutr Rev 2001 13 2 34 46

[B42] Stitt S An international perspective of food and cooking skills in education Brit Food J 1996 98 10 27 34 10.1108/00070709610153795

[B43] Stockley L Review of dietary intervention models for Black and minority ethnic groups 2009 http://www.food.gov.uk/multimedia/pdfs/reviewdietethnic1may09.pdf Retrieved from on November 28, 2009

